# Changes in the Bacterial Community of Soybean Rhizospheres during Growth in the Field

**DOI:** 10.1371/journal.pone.0100709

**Published:** 2014-06-23

**Authors:** Akifumi Sugiyama, Yoshikatsu Ueda, Takahiro Zushi, Hisabumi Takase, Kazufumi Yazaki

**Affiliations:** 1 Research Institute for Sustainable Humanosphere, Kyoto University, Uji, Kyoto, Japan; 2 Faculty of Bioenvironmental Science, Kyoto Gakuen University, Kameoka, Kyoto, Japan; Montana State Univeristy, United States of America

## Abstract

Highly diverse communities of bacteria inhabiting soybean rhizospheres play pivotal roles in plant growth and crop production; however, little is known about the changes that occur in these communities during growth. We used both culture-dependent physiological profiling and culture independent DNA-based approaches to characterize the bacterial communities of the soybean rhizosphere during growth in the field. The physiological properties of the bacterial communities were analyzed by a community-level substrate utilization assay with BioLog Eco plates, and the composition of the communities was assessed by gene pyrosequencing. Higher metabolic capabilities were found in rhizosphere soil than in bulk soil during all stages of the BioLog assay. Pyrosequencing analysis revealed that differences between the bacterial communities of rhizosphere and bulk soils at the phylum level; i.e., Proteobacteria were increased, while Acidobacteria and Firmicutes were decreased in rhizosphere soil during growth. Analysis of operational taxonomic units showed that the bacterial communities of the rhizosphere changed significantly during growth, with a higher abundance of potential plant growth promoting rhizobacteria, including *Bacillus*, *Bradyrhizobium,* and *Rhizobium*, in a stage-specific manner. These findings demonstrated that rhizosphere bacterial communities were changed during soybean growth in the field.

## Introduction

The rhizosphere is the small region around the roots, defined as “the zone of soil surrounding the root which is affected by it” [1], [2], where plants and millions of microbes interact with each other [3]. Rhizosphere microbes were shown to have intense activity and to be important for plant health and growth [4]. For example, mycorrhiza and rhizobia provide phosphorous and nitrogen, respectively, and microbes called plant-growth-promoting rhizobacteria (PGPR) exert both direct and indirect effects on plant growth, such as the prevention of colonization by pathogens and modulation of plant immunity [5]–[7]. These rhizosphere microbes are regarded as prominent components of sustainable agriculture that reduce the use of fertilizers and pesticides [8]. Plants have been shown to accommodate rhizosphere microbes by providing a platform and nutrients mainly at the root exudates, which account for up to 40% of photosynthates [9]–[12]. In addition to the climate and chemical properties of soils, resident plants exert influence on rhizosphere microbial communities. Microbial communities have been found to depend on the plant species grown in the same type of soil [13]–[16], demonstrating a tight interaction between plants and rhizosphere microbial communities [17].

Because rhizosphere microbial communities are important in plant growth and performance, these communities have been extensively studied using both culture-dependent and culture-independent methods [4], [17], [18]. Recent advances in next generation sequencing methods have enabled in-depth analyses of rhizosphere microbial communities. *Arabidopsis* root bacterial communities have been investigated comprehensively [19]–[21], and the analysis of soils collected under *Arabidopsis* plants in their natural habitats indicated possible interactions between *Arabidopsis* growth and the microbial communities in these soils [22]. However, despite increases in community-based analyses of rhizosphere bacterial communities [4], [17], it remains unclear how plant and bacteria communicate to form rhizosphere bacterial communities from ‘reservoir’ bulk soil. In particular, few studies have comprehensively analyzed rhizosphere microbial communities during growth using next generation sequencing [16], [23], [24] although growth-dependent analyses of rhizosphere microbial communities have been performed using methods such as automated ribosomal interspacer analysis (ARISA) and denaturing gradient gel electrophoresis (DGGE) [25]–[28].

Legume plants include important crop species such as soybeans (*Glycine max*), which supply nutrients rich in protein and oil for human consumption. Legume plants have been used to investigate plant-microbe interactions in the rhizosphere, due to their agricultural importance and ability to form symbiotic relationships with rhizobia and arbuscular mychorrhizal fungi (AMF) [29]–[31]. Genetic analysis using model legume plants such as *Lotus japonicus* and *Medicago truncatula* revealed pathways leading to symbiosis. Despite the large number of reports analyzing the components of legume-rhizobia and legume-AMF symbiosis, the broad range of rhizosphere microbial species in soil, which could affect legume interactions with rhizobia and AMF, have not been characterized in depth. The mechanisms underlying legume plant interactions with various soil microbes during growth in the field remain especially elusive. Understanding the composition of rhizosphere microbial communities during growth in the field could provide a basis for optimizing agricultural utilization of rhizosphere microbes. For example, DGGE showed that the composition of the soybean rhizosphere changed during growth with alterations in the relative contributions of various phyla, including Proteobacteria, Acidobacteria, Bacteroidetes, Nitrospirae, Firmicutes, Verrucomicrobia and Acidobacteria [27]. To enhance understanding of legume-microbe interactions in the field, and to obtain basic information on soybean rhizosphere bacterial communities for further research, bacterial communities of rhizospheres were analyzed together with bulk soil during soybean growth in a field in Kyoto Prefecture, Japan. The physiological properties of the bacterial communities were analyzed by community-level BioLog substrate utilization assays. In addition, the 16S ribosomal RNA (rRNA) gene, which is the most important target in the study of bacteria, was analyzed by PCR amplicon pyrosequencing. The results of this study suggest that plant growth could affect the composition of rhizosphere bacterial communities in addition to the seasonal effects.

## Materials and Methods

### Study site and sampling

Field experiments were conducted in an experimental field of Kyoto Gakuen University, Kameoka, Kyoto, Japan (N 34° 99′ 38′′, E 135° 55′ 14′′). This field has been maintained organically for more than 7 years, and sugar cane was grown there the previous year. Soybean seeds (*Glycine max* cv. Fukujishi) were purchased from Takii Shubyo (Kyoto, Japan) and sown on May 25, 2012. Plants were irrigated as needed, and emerging weeds were manually removed throughout the crop season on a weekly basis. Bulk soil samples were collected before sowing the seeds. Rhizosphere and bulk soil samples were collected on July 9, 2012 (at the beginning of flowering, R2), on August 25, 2012 (after the seeds matured, but pods were still green, R6), and on September 24, 2012 (at full maturity, when the pods were dry, R8). Bulk soil, defined a soil that does not adhere to plant roots, was obtained at least 20 cm from the plants. Bulk soils from five different spots were combined into one sample. Rhizosphere soil, defined as soil that adheres to the plant root after gentle shaking [32], was obtained from five plants, using sterile brushes, and combined into one sample. Both rhizosphere and bulk soil samples were immediately transferred to the laboratory in a cool container (0–10°C) within 2 hours. Each sample was homogenized and passed through a 1 mm sieve, and 1.5 g of each sample was immediately used for the BioLog substrate utilization assay. The remaining soil was kept at −30°C until subsequent DNA extraction. The chemical properties of the soil at initial stage were analyzed using standard methods in the soil analysis laboratory of Otsuka Agritechno Co. Ltd. (Tokyo, Japan). The soil was found to have an electrical conductivity of 0.28 dS/m, a pH of 6.7, and to contain 0.5 ppm NH_4_^+^, 30 ppm, NO_3_^−^, 5.7 ppm K, 3.2 ppm S, 3.72 ppm Ca, 7.6 ppm Mg, 0.02 ppm B, 0.58 ppm Fe, 0.01 ppm Cu, 0.03 ppm Zn, and 1.6 ppm Cl. Neither P nor Mn was detected. Detection limit for P and Mn was 0.02 and 0.0003 ppm, respectively. No fertilizers and pesticides were applied during the growth season.

### DNA extraction and real time PCR to quantify total bacteria

DNA was extracted from 0.5 g soil using the Soil DNA Isolation Kit (Mo Bio, Carlsbad, CA) according to the manufacturer's protocol. Extracted DNA was quantified using the Qubit Quantification Platform dsDNA HS Assay Kit (Invitrogen, Carlsbad, CA) and stored at −30°C until use.

For the real time PCR to quantify the total bacterial communitieswe used 16S rRNA gene-specific primers (968F and 1401R) essentially as described previously [33]. Amplification reaction was carried out using Thunderbird SYBR qPCR mix (Toyobo, Osaka, Japan) according to the manufacture's protocol. Amplification reactions were as follows: 2 min at 95°C, 40 cycles of 15 sec at 95°C, 30 sec at 50°C, 30 sec at 72°C.

### Barcoded pyrosequencing and sequence analysis

For the amplicon pyrosequencing the 16S rRNA gene was amplified using the barcorded primers; 27F, 5′-gcctccctcgcgccatcagnnnnnnnnnnnnAGAGTTTGATCMTGGCTCAG-3′ and 388R, 5′-gccttgccagcccgctcagTCTGCTGCCTCCCGTAGGAGT-3′ [34]–[36]. The lowercase sequences of each primer are necessary adapters for binding and amplification during pyrosequencing. The uppercase sequences targeted the conserved regions of the 16S rRNA gene [37], [38], and the “n” repeats represent unique barcodes, which allow the sorting and assignment of each individual sequence read. The barcode sequences were provided by Operon Co Ltd., and are listed in Table S1. Each 25 µL PCR mixture contained 20 ng soil DNA and 1 U Tks Gflex DNA polymerase (Takara, Japan). PCR amplification was performed in a thermal cycler (iCycler, BioRad, Hercules, CA), with the protocol consisting of an initial denaturation at 94°C for 1 min, followed by 30 cycles of denaturation at 98°C for 10 sec, annealing at 55°C for 15 sec, and extension at 68°C for 30 sec. PCR amplicons were verified by electrophoresis on 1.2% agarose gels. A total of nine PCR amplicons (three PCR replicates x three different DNA samples) were combined for each barcode, and bands were extracted using the Wizard SV Gel and PCR Clean-Up System (Promega, San Luis Obispo, CA) according to the manufacturer's protocol. The DNA concentration of each purified PCR product was measured using the Qubit Fluorometer with Quant-iT dsDNA BR Assay Kit (Invitrogen) according to the manufacturer's protocol. Equal amounts of PCR amplicons harboring 21 different barcode sequences (7 samples×3 replicates) were combined into a single tube, and ethanol-precipitated. Dried DNA samples were sent to the next-generation sequencing laboratory of Operon Co., Ltd., and analyzed using the GS Junior Titanium System (Roche, Basel, Switzerland).

Sequence reads were imported into Mothur v. 1.29.0 (http://www.mothur.org) for operational taxonomic unit (OTU) generation and classification [39]. All sequence data was de-noised through the default parameters of Mothur (shhh.flows), and de-noised sequence reads were trimmed using the following criteria (trim.seqs): no more than two mismatches for the forward primer sequence, no more than one mismatch in the barcode sequence, no homopolymeric sequences of more than eight nucleotides, a read length of more than 200 bases, and trimming to a common length of 200 base pairs. A bacterial 16S rRNA reference alignment was exported from SILVA [40]. Chimera sequences were detected using the chimera.uchime command in Mothur [41] and were removed (remove.seqs) from further analysis. Sequences were aligned using the Needleman-Wunsch algorithm, pairwise genetic distances were calculated (calc  =  onegap, countends  =  T), and sequences were clustered into OTUs using the furthest neighbor algorithm (cutoff = 0.03). All 454 pyrosequencing reads have been deposited into the DDBJ Sequence Read Archive under accession number DRA001076 [42]. Statistical analyses were performed using PC-ORD Software v. 6.0, as described [22], [35], [43] and the R statistical package (R Development Core Team).

### Microbial community analysis BioLog EcoPlates

BioLog analysis was performed as described [22]. Briefly, 1.5 g aliquots of three soil samples were each added to 15 ml sterile saline solution (0.87% w/v NaCl), and diluted 1∶10,000 with the same solution. The diluted solution (150 µL) was added to each well of the BioLog EcoPlate (Biolog, CA), followed by incubation at 25°C for 96 h. Absorbance at 595 nm was recorded with Spectraflour plus (TECAN), and data were analyzed using the average well color development (AWCD) method [44].

## Results

### Soil sampling and community-level physiological profiling

**Figure 1:**
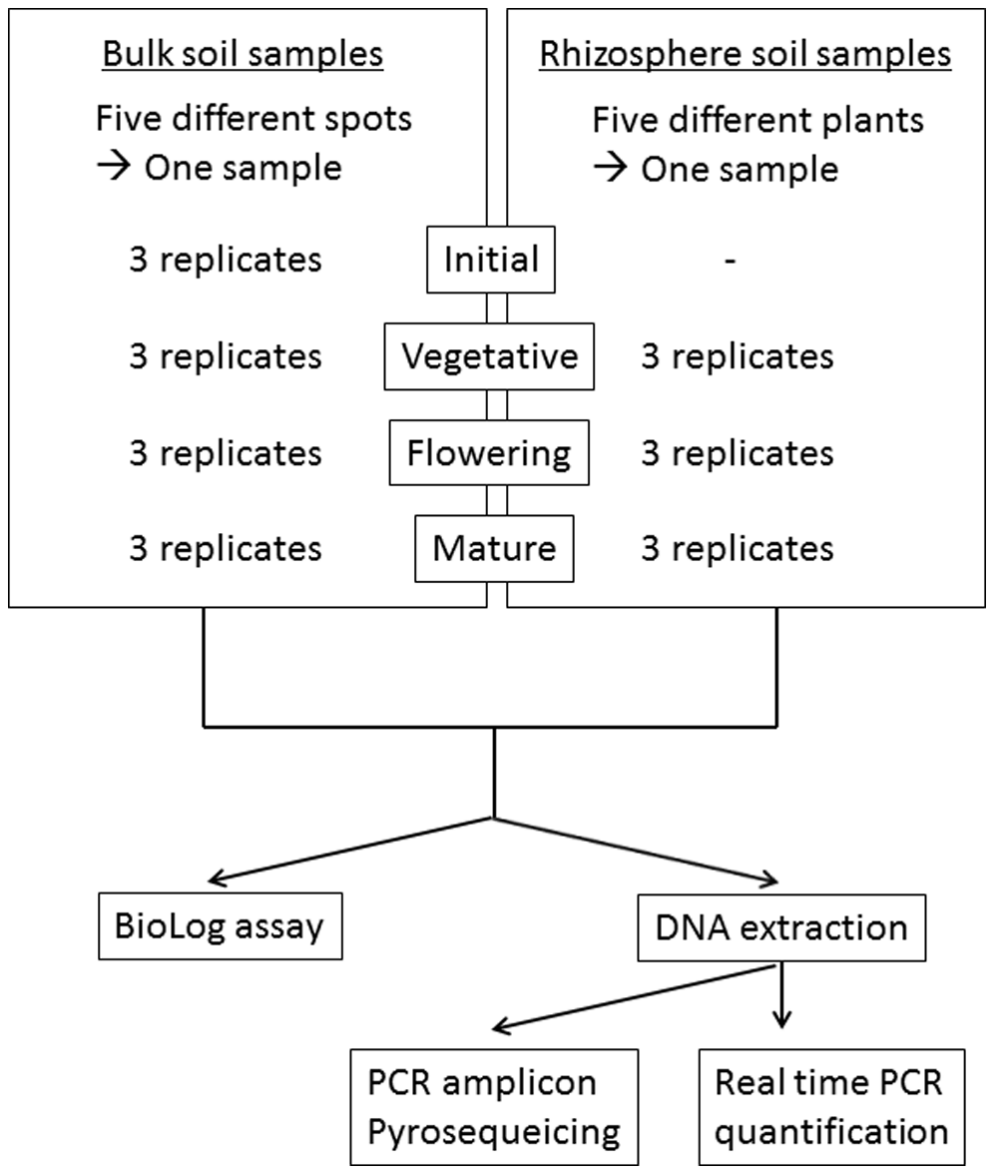
Bulk and rhizosphere sample preparation.

**Figure 2:**
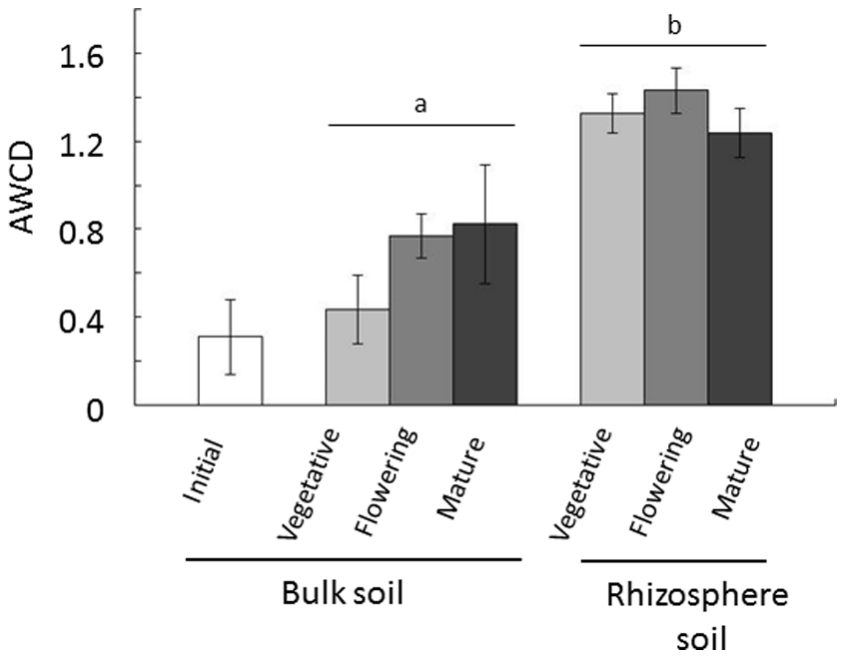
Community level physiological profiling. The BioLog substrate utilization assay was performed to generate community-level substrate utilization profiles. Average well color development (AWCD) after a 96-hour incubation was recorded. Values are mean ± SD (n = 3). The effect of growth stage and interaction between both factors (growth stage and compartment) were not significant in two-way ANOVA, but significant difference was observed between bulk and rhizosphere soil, as shown by the different letters (p<0.05).

Soybeans were grown from May to September 2012 in a field in Kyoto Prefecture, Japan. Soil samples were obtained during the initial stage (before the seeds were sown), the vegetative stage (beginning of flowering, R2), the flowering stage (after seeds became mature, but the pods were still green, R6), and in the mature stage (pods were dry, R8), as described in the Materials and Methods section. A flow chart is also shown in Figure 1. The BioLog substrate utilization assay was performed on the day of sampling to avoid changes in bacterial communities during storage of the soil. The BioLog assay was originally developed to identify microbial isolates based on their substrate utilization profiles, and is now widely used to obtain community-level substrate utilization profiles [22], [44]–[47]. Color intensity was determined by calculating the average well color development (AWCD) on each plate. The AWCD of rhizosphere soil was 1.5 to 3-fold higher than that of bulk soil throughout soybean growth when same amount of soil was used (Figure 2), indicating that tested metabolic capabilities of rhizosphere soil were higher than bulk soil and that the physiological profile of the two sets of soil samples differed significantly. Overall, most of the substrates were highly metabolized by rhizosphere soils (Table S2). Total bacterial communities were also quantified using real time PCR, demonstrating that 2.0×10^8^ to 5.2×10^8^ gene copy number of 16S rRNA were observed in soils (Table S3). When BioLog data were normalized with 16S rRNA gene, higher activities were observed in rhizosphere soil than bulk soils at vegetative and flowering stages, but not in mature stage. Significantly higher activities were observed in mature soils of both bulk and rhizosphere, compared with vegetative and flowering stages (Figure S1).

### Phylum-level analysis of bacterial communities during soybean growth

DNA was extracted from both bulk and rhizosphere soil and stored at −30°C. PCR was subsequently performed, and the amplicons were sequenced using the GS Junior Titanium System (Roche). A total of 62,828 sequences from 21 samples were obtained from the pyrosequencing reactions (7 different soil types in triplicate, Table S4). Sequences were analyzed using Mothur v.1.29.0 [39] and The Ribosomal Database Project Pyrosequencing Pipeline.

**Figure 3:**
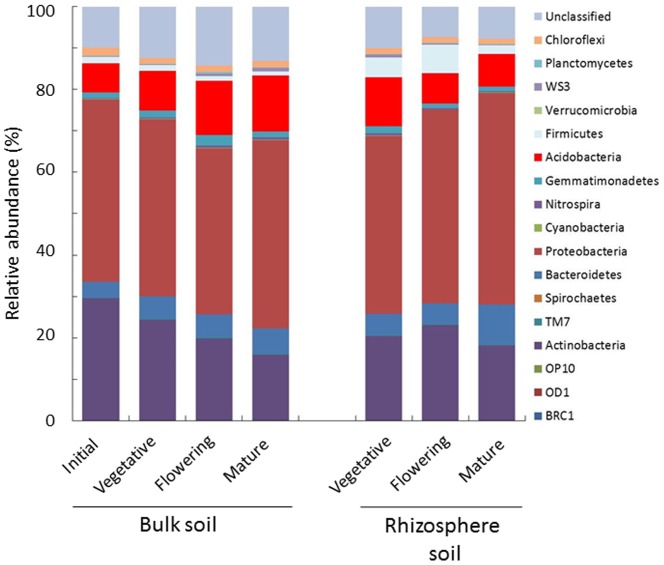
Relative abundance of major bacterial phyla present in bulk and rhizosphere soil during soybean growth, as revealed by pyrosequencing. Each bar represents the average relative abundance of triplicates.

All sequences could be classified into 17 phyla. Proteobacteria was the dominant phyla in all soils, followed by Actinobacteria and Chloroflexi (Figure 3 and Figure S2). Rhizosphere soil showed changes distinct from bulk soil during soybean growth, with Proteobacteria increasing from 42.8±2.2% in the vegetative stage to 51.1±3.9% in the mature stage, while Acidobacteria decreased from 11.8±2.9% to 7.8±1.6% and Firmicutes decreased from 4.8%±2.2 to 2.1±0.6%. In bulk soil, however, Acidobacteria was increased from 7.1±0.8% to 13.5±1.2%, while Actinobacteria was decreased, from 29.4±0.8% to 16.0±1.2%, during soybean growth.

### OTU-based analysis of bacterial communities during soybean growth

**Figure 4:**
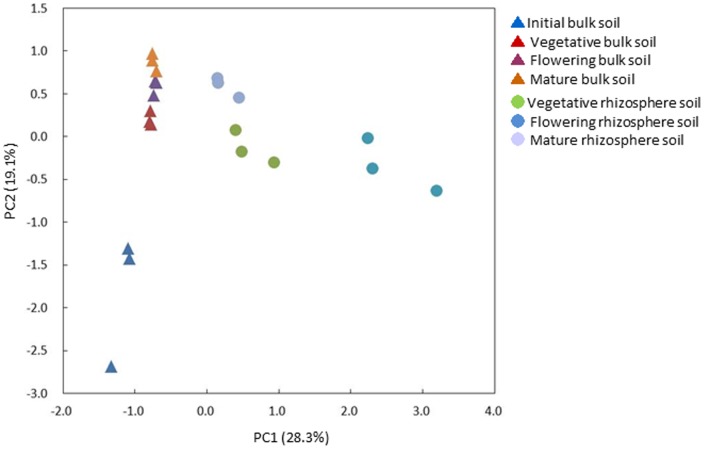
Principal component analysis of the relative abundance of OTUs.

Principal Component Analysis (PCA) was performed with the relative abundance values of OTUs used to compare bacterial communities at each stage (Figure 4). The bacterial community structures of bulk soil in the vegetative, flowering, and mature stages differed from those of initial bulk soil, but the former three communities clustered together, suggesting that relatively small changes occurred in the bacterial communities of bulk soil during soybean growth. In contrast, the communities of rhizosphere soil differed during all four stages (Figure 4), suggesting that rhizosphere bacterial communities that formed during the vegetative stage were further modified during growth. To characterize the changes in the bacterial communities of bulk and rhizosphere soil during soybean growth, the relative abundances of OTUs were compared during growth, with a list of the 1,000 most abundant OTUs shown in Table S5. The most abundant OTU was found to be *Bradyrhizobium* (Rank 1), followed by Steroidobacter (Rank 2), and unclassified bacteria (Rank 3).

**Figure 5:**
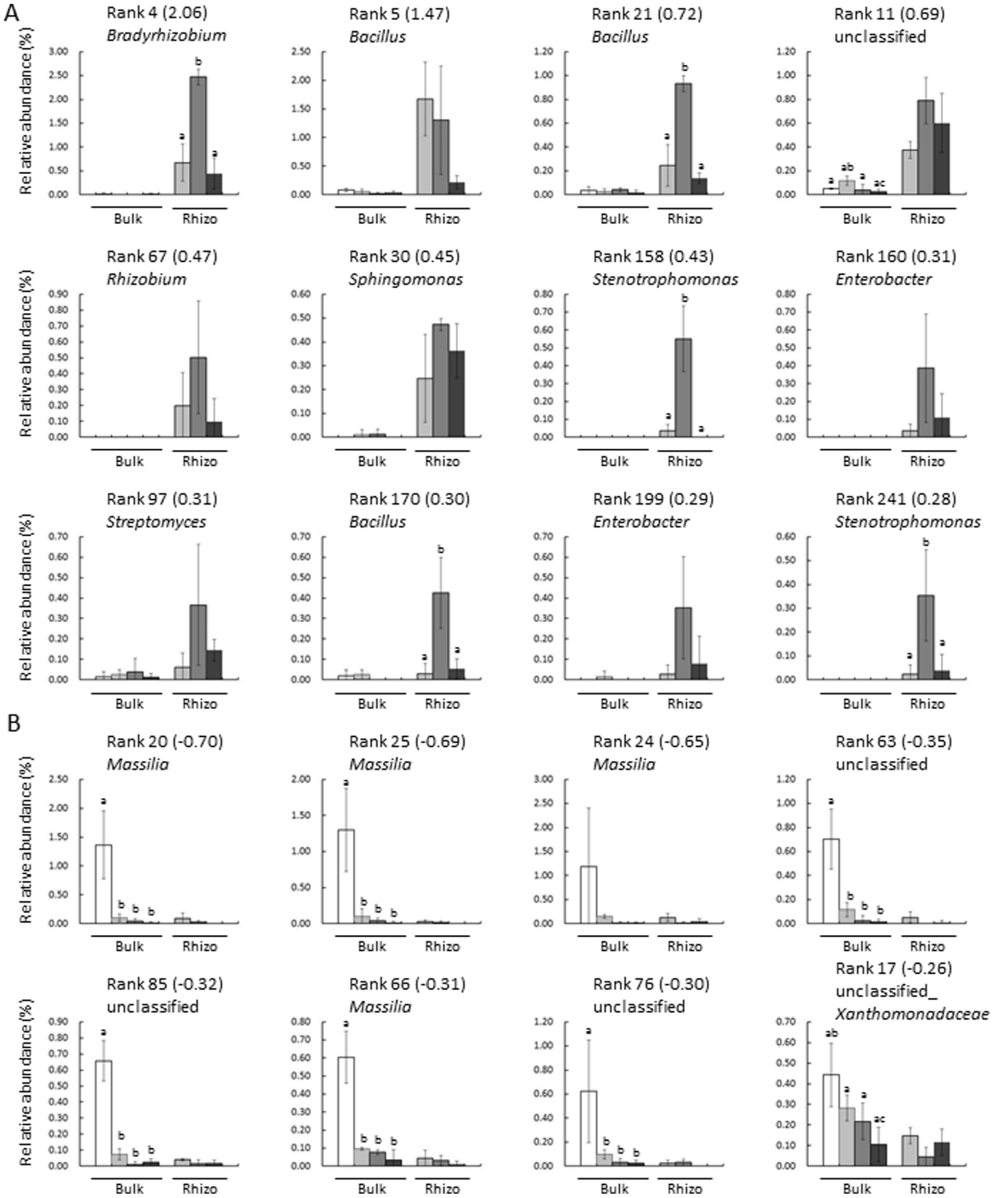
Relative abundance of OTUs with high absolute loading on Principal Component 1 (PC1). (A) OTUs with high positive loading on PC1; (B) OTUs with high negative loading on PC1. White bars represent bulk soil and black bars represent rhizosphere soil. The relative abundance was calculated using the data from pyrosequencing analysis. The numbers in parentheses are loading values. □; initial soil, ▪; vegetative stage, ▪; flowering stage, ▪; mature stage. Values are mean ± SD (n = 3), with significant differences by Tukey's HDS test (P<0.05) indicated.

PCA analysis showed that the first principal component (PC1) (28.3%) appeared to represent the difference in bacterial communities between bulk and rhizosphere soil as well as the changes of rhizosphere bacterial communities during growth, whereas PC2 (19.1%) represented the difference in bacterial communities between initial soil and soil after soybean growth. To characterize the changes in bacterial communities during growth in detail, 20 OTUs that showed high absolute loading on PC1 were selected (12 positive and 8 negative), and their relative abundance was compared (Figure 5). Positive (+) and negative (−) loadings correlate positively and negatively with axis, respectively. OTUs with high positive loading on PC1 were more abundant in rhizosphere than in bulk soil. *Bradyrhizobium* (rank 4), *Bacillus* (rank 21 and 170), and *Stenotrophomonas* (rank 158 and 241) were highly increased during the flowering stage. In contrast, OTUs with high negative loading were more abundant in initial bulk soil and less abundant in both bulk and rhizosphere soil after soybean growth. Most of these OTUs were found to be *Massilia* (Figure 5B), a group of Gram-negative Betaproteobacteria widely found in soils, while their effects on resident plant species have not yet been reported[21], [48].

**Figure 6:**
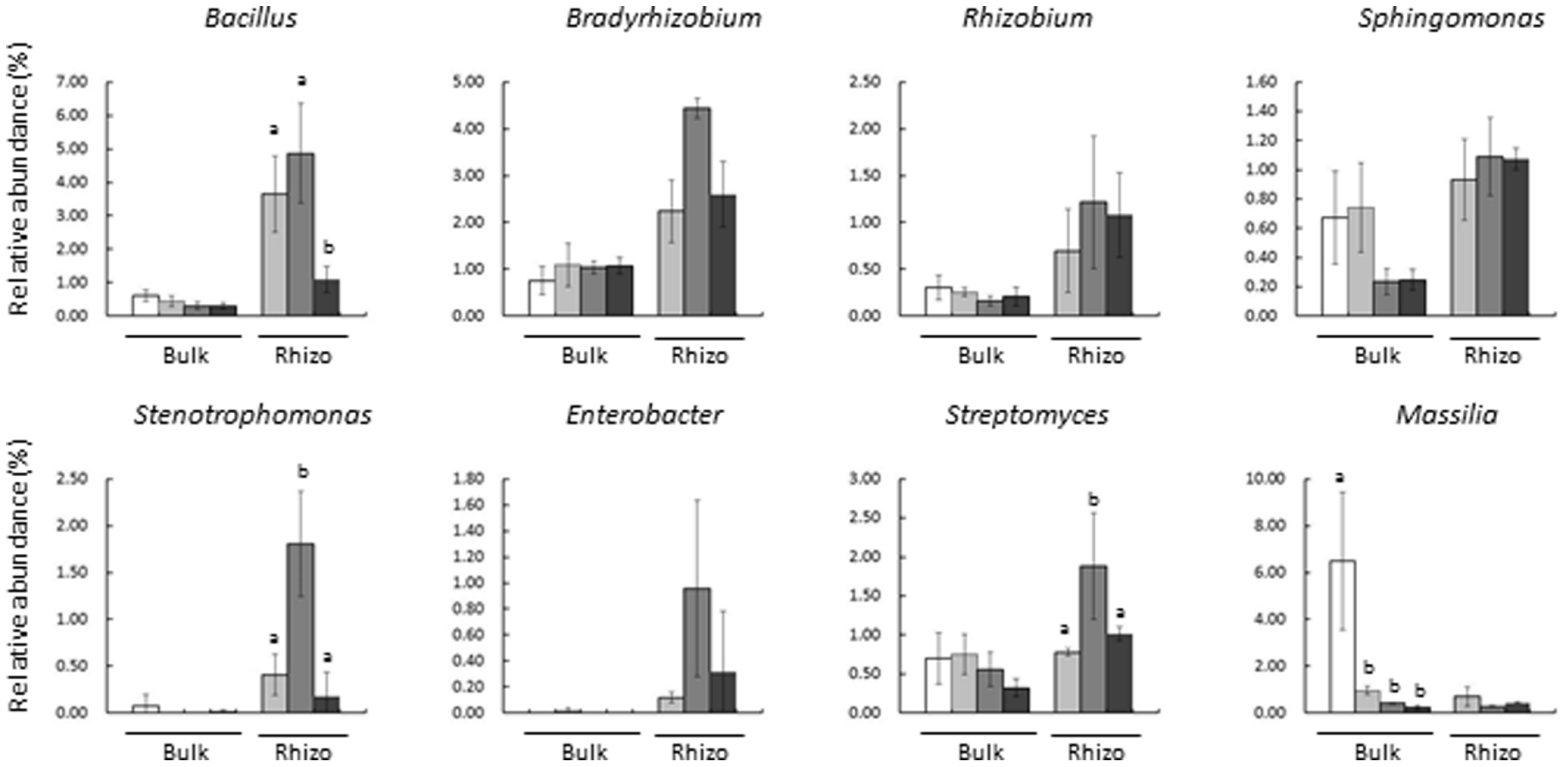
Relative abundance of eight different genera during soybean growth. The relative abundance of the eight genera shown in Figure 4 was compared. □; initial soil, ▪; vegetative stage, ▪; flowering stage, ▪; mature stage. Values are mean ± SD (n = 3), with significant differences by Tukey's HDS test (P<0.05) indicated.

The pyrosequencing of 16S rDNA reveals bacterial communities at the genus level; therefore, all OTUs found to belong to each genus (Figure 5) were combined, and the relative abundance of these genera was compared during growth (Figure 6). *Bacillus* was one of the most abundant genera in the soybean field, with a relative abundance in rhizosphere soil higher during the vegetative and flowering stages than during the mature stage. The relative abundances of *Stenotrophomonas* and *Strepromyces* in rhizosphere soil were significantly higher during the flowering stage than during either the vegetative or mature stage. In contrast, the relative abundance of *Massilia* was highest in the initial bulk soil, accounting for up to 6% of the bacterial community in these samples (Figure 6). The relative abundance of these genera showed similar patterns as the representative OTUs in Figure 4, suggesting that a large proportion of community changes occurred at the genus level.

## Discussion

Plants have been found to influence rhizosphere bacterial communities, which are important for crop growth and yield [4], [49]. In this study, the rhizosphere bacterial communities of soybeans grown in the field were analyzed using both culture-dependent physiological profiling and culture independent 16S rRNA metagenomics. High numbers of bacteria have been reported to flourish in the rhizosphere, due mostly to the supply of nutrients and the platform supplied by resident plants [3], [17], [50]. Using BioLog substrate utilization assays, we found that tested metabolic capabilities of rhizosphere soil were higher than bulk soil during soybean growth, and rhizosphere soil had higher microbiological activity in the vegetative stage (Figure 2). These results suggest that soybeans affected rhizosphere bacterial communities within 6 weeks after sowing (vegetative stage), with further alterations possibly occurring during later growth stages to maintain physiologically active rhizosphere bacterial communities.

A metagenomic 16S rRNA amplicon approach was employed to characterize the bacterial communities of both bulk and rhizosphere soil during soybean growth. Pyrosequencing of PCR amplicons and sequence analyses using Mothur v. 1.29.0 [39] revealed that Proteobacteria was the most abundant phylum in all soil types, with changes in bacterial communities at the phylum level during soybean growth occurring in both rhizosphere and bulk soil (Figure 3). Of the Proteobacteria, the Alphaproteobacteria were the most abundant, followed by Beta-, Gamma-, and Deltaproteobacteria in all soil types (Figure S3). The relative abundance of the Alpha- and Gammaproteobacteria, as well as their ratio, was found to be altered drastically by nitrogen fertilization and soybean nodulation [51]; however, we found only minor changes in these two classes in both bulk and rhizosphere soil during soybean growth. OTU-based analyses revealed that rhizosphere bacterial communities changed as soybean growth stage changed, suggesting the stage-specific formation of unique rhizosphere bacterial communities (Figure 4). In contrast, bulk soil bacterial communities showed small changes during plant development, but differed markedly from initial stage bulk soil bacterial communities. The latter was likely due to seasonal changes, as well as distant effects from resident plant species.

It has been hypothesized that resident plant species accommodate a specific set of rhizosphere bacterial communities, both to optimize growth and to prevent infection by pathogens [4]. In this study, *Bradyrhizobium* was shown to be abundant in rhizosphere soil, presumably due to the chemotaxis of *Bradyrhizobium* to the root exudates of soybean and proliferation around the roots [52]. Other genera that are highly abundant in rhizosphere soil (Figures 5 and 6) also contain potential PGPRs. For example, various species of *Bacillus* have been associated with systemic resistance to pathogens [53], phosphorus solubilization, and the production of antibiotics [54], [55]. Furthermore, *Stenotrophomonas maltophilia* was found to reduce nematode densities in soils [56] and *Enterobacter cloacae* to produce indole-3-acetic acid and solubilize phosphate [57], [58]. In addition, *Sphingomonas* sp. was found to produce indole-3-acetic acid [59] and *Streptomyces* sp. to reduce the infection of pine roots by *Fusarium* and *Armillaria* [60], although both these genera also contain plant pathogens, including *Sphingomonas melonis* [61] and *Streptomyces scabies* [62], [63]. We found that several species of these potential PGPRs were elevated in the rhizosphere of soybean at growth stage-specific manner. This may be due to changes in soybean root exudates during growth, inasmuch as the profiles of root exudates and rhizosphere microbes have been reported to correlate during the growth of *Arabidopsis* [23], [24].

In conclusion, this study revealed the changes that occur in the rhizosphere bacterial communities of soybeans grown in the field. Physiological activities measured by BioLog assay suggested that metabolic capabilities of rhizosphere soil were higher than bulk soil during the growth season. Rhizosphere bacterial communities were changed during soybean growth, in a manner distinct from bacterial communities in bulk soil, and rhizosphere bacterial communities contained potential PGPR genera that are both highly abundant and growth stage-specific. Changes observed in rhizosphere bacterial communities are in part due to the seasonal and surface effects as for bulk soil communities, but substantial changes observed for rhizosphere communities suggest the influence from soybean growth. Further studies on both metabolic activities of soybean such as root exudation and the physiological functions of these rhizobacteria on plant growth are warranted to elucidate the reciprocal interactions between plants and rhizosphere microbes in the fields for better utilization of rhizosphere bacteria for sustainable agriculture.

## Supporting Information

Figure S1 **Community level physiological profiling normalized in 16S rRNA gene.** The BioLog substrate utilization assay was performed to generate community-level substrate utilization profiles. Average well color development (AWCD) after a 96-hour incubation was recorded, and normalized with 16S rRNA gene. Values are mean ± SD (n = 3). Different letters indicate significant differences (P<0.05) by one-way ANOVA with Tukey's HSD test.
(TIF)

Figure S2 **Relative abundance of each bacterial phylum.** The relative abundance of each phyla was compared. □; initial soil, ▪; vegetative stage, ▪; flowering stage, ▪; mature stage. Values are mean ± SD (n = 3)
(TIF)

Figure S3 **Relative abundance of each class of Proteobacteria.**
(TIF)

Table S1 **Barcode sequences used for pyrosequencing primers.**
(DOCX)

Table S2 **Substrate utilization indicated by color development in Biolog Eco Plates.**
(XLSX)

Table S3 **Gene copy numbers of 16S rRNA in soil.**
(DOCX)

Table S4 **Number of sequences reads used for analysis.**
(DOCX)

Table S5 **The 1,000 most abundant OTUs found in the experimental soybean field.**
(XLSX)
