# Supplementary figures and images for: Changes in the Bacterial Community of Soybean Rhizospheres during Growth in the Field
Source: PLoS One. 2014 Jun 23;9(6):e100709. doi: 10.1371/journal.pone.0100709 (PMC4067361; doi:10.1371/journal.pone.0100709)

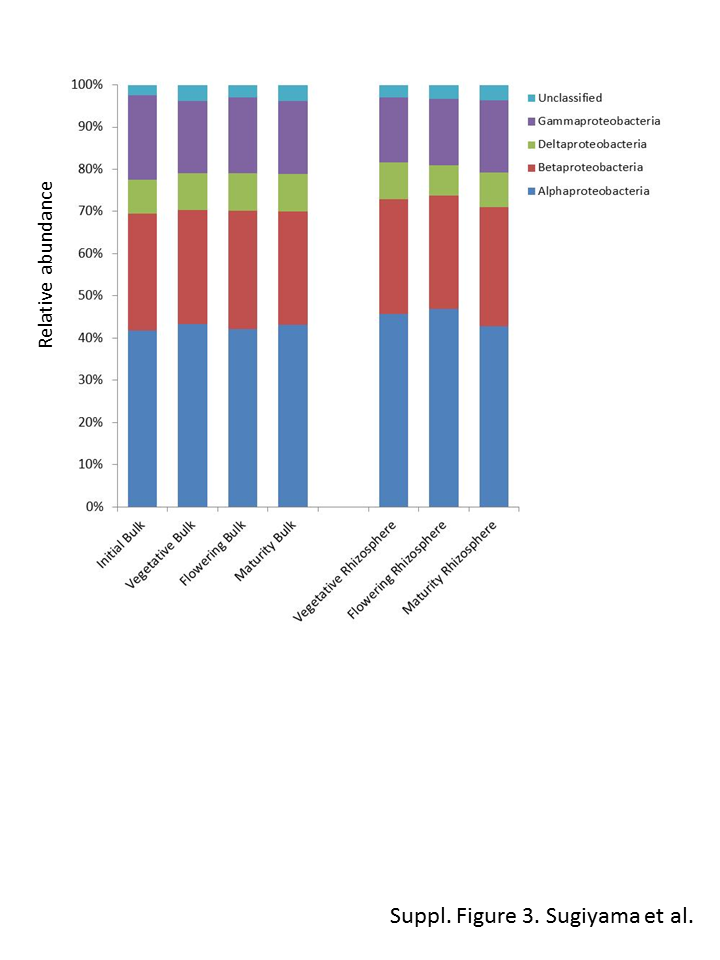

Supplement: Figure S3 — Relative abundance of each class of Proteobacteria. (TIF) [file pone.0100709.s003.tif]
