# Supplementary material for: Changes in the Bacterial Community of Soybean Rhizospheres during Growth in the Field
Source: PLoS One. 2014 Jun 23;9(6):e100709. doi: 10.1371/journal.pone.0100709 (PMC4067361; doi:10.1371/journal.pone.0100709)
Supplement: Table S1 — Barcode sequences used for pyrosequencing primers. (DOCX) [file pone.0100709.s004.docx]

Table S1. Barcode sequences used for pyrosequencing primers

Sample Sequence

Initial-1 ACGAGTGCGT

Initial-2 ACGCTCGACA

Initial-3 AGACGCACTC

Vegetative bulk-1 CGTGTCTCTA

Vegetative bulk-2 CTCGCGTGTC

Vegetative bulk-3 TCGTCGCTCG

Vegetative rhizosphere-1 AGCACTGTAG

Vegetative rhizosphere-2 ATCAGACACG

Vegetative rhizosphere-3 ATATCGCGAG

Flowering bulk-1 ATACGACGTA

Flowering bulk-2 TCACGTACTA

Flowering bulk-3 CGTCTAGTAC

Flowering rhizosphere-1 TGATACGTCT

Flowering rhizosphere-2 CATAGTAGTG

Flowering rhizosphere-3 CGAGAGATAC

Mature bulk-1 CGTAGACTAG

Mature bulk-2 TACGAGTATG

Mature bulk-3 TACTCTCGTG

Mature rhizosphere-1 TCTACGTAGC

Mature rhizosphere-2 TGTACTACTC

Mature rhizosphere-3 ACGACTACAG
