# Supplementary material for: Changes in the Bacterial Community of Soybean Rhizospheres during Growth in the Field
Source: PLoS One. 2014 Jun 23;9(6):e100709. doi: 10.1371/journal.pone.0100709 (PMC4067361; doi:10.1371/journal.pone.0100709)
Supplement: Table S3 — Gene copy numbers of 16S rRNA in soil. (DOCX) [file pone.0100709.s006.docx]

Table S3. 16S rRNA gene copy number in soils.

Gene copy number

Initial 4.6 x 10^8^ ± 5.6 x 10^7^

Vegetative bulk 3.4 x 10^8^ ± 1.3 x 10^8^

Vegetative rhizosphere 4.6 x 10^8^ ± 1.1 x 10^8^

Flowering bulk 5.2 x 10^8^ ± 1.5 x 10^8^

Flowering rhizosphere 4.9 x 10^8^ ± 2.2 x 10^8^

Mature bulk 2.0 x 10^8^ ± 7.0 x 10^6^

Mature rhizosphere 2.5 x 10^8^ ± 9.1 x 10^7^
