# Supplementary material for: Changes in the Bacterial Community of Soybean Rhizospheres during Growth in the Field
Source: PLoS One. 2014 Jun 23;9(6):e100709. doi: 10.1371/journal.pone.0100709 (PMC4067361; doi:10.1371/journal.pone.0100709)
Supplement: Table S4 — Number of sequences reads used for analysis. (DOCX) [file pone.0100709.s007.docx]

Table S4. Numbers of reads used for analysis

Sample Raw Filtered Final (Chimera checked)

Initial-1 2069 1864 1555

Initial-2 2137 1904 1676

Initial-3 2654 2364 2017

Vegetative bulk-1 3550 3136 2683

Vegetative bulk-2 3252 2810 2446

Vegetative bulk-3 2523 2221 1990

Vegetative rhizosphere-1 3272 2868 2308

Vegetative rhizosphere-2 2760 2438 2102

Vegetative rhizosphere-3 2524 2202 1901

Flowering bulk-1 3686 3276 2820

Flowering bulk-2 3054 2740 2338

Flowering bulk-3 3627 3244 2850

Flowering rhizosphere-1 2477 2222 1793

Flowering rhizosphere-2 2355 2096 1710

Flowering rhizosphere-3 2575 2279 1885

Mature bulk-1 2792 2508 2206

Mature bulk-2 3228 2881 2528

Mature bulk-3 3464 3100 2703

Mature rhizosphere-1 3269 2949 2523

Mature rhizosphere-2 3709 3342 2794

Mature rhizosphere-3 3851 3492 2837
